# Long-term unprocessed and processed red meat consumption and risk of chronic obstructive pulmonary disease: a prospective cohort study of women

**DOI:** 10.1007/s00394-018-1658-5

**Published:** 2018-03-12

**Authors:** Joanna Kaluza, Holly Harris, Anders Linden, Alicja Wolk

**Affiliations:** 1 Unit of Nutritional Epidemiology, Institute of Environmental Medicine, Karolinska Institutet, 171-77 Stockholm, Sweden; 2 Nutrition Research Laboratory, Department of Human Nutrition, Warsaw University of Life Sciences, SGGW, 159C Nowoursynowska Str., 02-776 Warsaw, Poland; 3 Program in Epidemiology, Division of Public Health Sciences, Fred Hutchinson Cancer Research Center, Seattle, WA USA; 4 Unit for Lung and Airway Research, Institute of Environmental Medicine, Karolinska Institutet, Stockholm, Sweden; 5 Lung Allergy Clinic, Karolinska University Hospital New Karolinska Solna, Stockholm, Sweden; 6 Unit of Orthopedics, Department of Surgical Sciences, Uppsala University, Uppsala, Sweden

**Keywords:** Chronic obstructive pulmonary disease, Diet, Epidemiology, Processed meat, Red meat, Prospective cohort study

## Abstract

**Purpose:**

Limited studies have examined red meat consumption in relation to risk of chronic obstructive pulmonary disease (COPD), and none have examined the impact of long-term diet on COPD risk. We sought to investigate the association between long-term red meat consumption and risk of COPD.

**Methods:**

The population-based prospective Swedish Mammography Cohort included 34,053 women, aged 48–83 years, followed for the current analyses from 2002 to 2014. Unprocessed and processed red meat consumption was assessed with a self-administered questionnaire in 1987 and 1997. Cox proportional hazard models were used to estimate hazard ratios (HRs) and 95% confidence intervals (CIs).

**Results:**

Over a mean follow-up of 11.6 years (2002–2014; 393,831 person-years), 1488 COPD cases were ascertained via linkage to the Swedish health registers. A positive association between long-term processed red meat (average from 1987 to 1997) and risk of COPD was observed. In contrast, no association was observed with unprocessed red meat with corresponding HRs of 1.36 (95% CI 1.03–1.79) for processed and 0.87 (95% CI 0.74–1.02) for unprocessed red meat among women who consumed ≥ 50 g/day compared to < 25 g/day. The observed association with processed meat was confined to ex-smokers (*P* for interaction = 0.30); women consuming of ≥ 50 g/day of processed meat had a 2.3-fold (95% CI 1.24–4.12) higher risk of COPD than those consuming < 25 g/day. No similar associations were observed among current or never smokers.

**Conclusion:**

In this prospective cohort of women with moderate red meat consumption, long-term processed red meat consumption was associated with an increased risk of COPD particularly among ex-smokers.

**Electronic supplementary material:**

The online version of this article (10.1007/s00394-018-1658-5) contains supplementary material, which is available to authorized users.

## Introduction

According to World Health Organization chronic obstructive pulmonary disease (COPD) is estimated to be the third leading cause of death worldwide [1]. Important risk factors for COPD include genetic predisposition, environmental pollution, and lifestyle factors including tobacco smoking, which is the most common cause of COPD in developed countries.

The results of recent systematic reviews [2] and meta-analyses indicate that high consumption of red meat and/or processed red meat is associated with increased risk of all-cause mortality [3] as well as major chronic diseases, including cardiovascular disease [4], stroke [5], some cancers [6, 7], and diabetes [8].

Two previous prospective studies have examined baseline processed red meat consumption in relation to COPD [9, 10], and only one study has assessed the association both unprocessed as well as processed meat [11]. Results of these studies indicated that baseline processed [9–11] but not unprocessed [11] red meat consumption may be associated with risk of COPD among smokers. However, no previous studies have assessed the risk of COPD in relation to long-term red meat consumption.

Therefore, in the large population-based prospective Swedish Mammography Cohort we evaluated the associations between long-term red meat consumption, separately for unprocessed and processed red meat, with COPD incidence in women, and assessed whether smoking status, the main predictor of COPD, modified the association.

## Methods

### Study population and design

The Swedish Mammography Cohort (SMC) was established between 1987 and 1990. All women born between 1914 and 1949 and residing in Västmanland and Uppsala counties in central Sweden were invited to participate in the study and asked to complete a questionnaire (response rate 74%). In 1997 the women who were still alive were sent a second questionnaire to update the information on food consumption and lifestyle factors (response rate 70%). In the present study, the 1997 questionnaire was considered baseline because information on some COPD risk factors such as smoking habits and physical activity were not obtained on from first questionnaire in 1987–1990. Due to a potential under-diagnosis of COPD in the first years of follow-up (1998–2001), we introduced a 4-year lag period and the study period for the main analysis started on 1 January 2002 (details in the “Case ascertainment” section).

Of the 39,227 women who returned a questionnaire in 1997, we excluded those with a missing or incorrect national identification number (*n* = 243), with implausible values for total energy intake in 1997 (> 3 standard deviations from the mean value for log-transformed energy, *n* = 491), and those with missing data for unprocessed or processed red meat consumption in 1987–1990 or 1997 (*n* = 1395). We also excluded women who were diagnosed with cancer before baseline (other than non-melanoma skin cancer, *n* = 1717), those who were diagnosed with COPD before baseline or during the 4-year lag period (*n* = 439) or who died during the 4-year lag period (*n* = 889). After these exclusions, a total of 34,053 women remained and were followed to the date of the first COPD diagnosis, death, or the end of follow-up (31 December 2014).

Completion and return of the self-administered questionnaire constituted the participants’ informed consent. The study was reviewed and approved by the Central Ethical Review Board in Stockholm, Sweden (decision no. 2016/2034-31/1) and was conducted fully in line with the Helsinki Declaration.

### Dietary assessment

In 1987–1990 and 1997 diet was assessed using a food frequency questionnaire (FFQ) which included 67 and 96 food items, respectively. Participants were asked to indicate how often, on average, they had consumed various foods over the past year by using predefined frequency categories from “never/seldom” to “3 or more times per day”. We categorized meat consumption into unprocessed and processed red meat; where unprocessed red meat included three food items (pork, beef/veal and minced meat), while processed red meat included four food items (sausages/hot dogs, cold cuts such as ham/salami, blood pudding, and liver pate). The frequencies of red meat consumption were converted to grams per day by multiplying the frequency of consumption of each food item by appropriate age-specific portion sizes. The long-term consumption of red meat was estimated for each participant by calculating the average unprocessed and processed red meat consumption from the questionnaires in 1987–1990 and 1997.

The baseline FFQ has been validated in a sample of 129 women randomly chosen from the cohort. The Spearman correlation coefficients between the baseline FFQ and the average of four 1-week weighted diet records ranged from 0.3 to 0.7 for red meat and processed meat items [2].

### Covariates

Using data collected in 1997, we assessed dietary patterns based on two measurements of diet quality, that is, Recommended Food Score (RFS) and modified Non-Recommended Food Score (Non-RFS), which have been described previously [11, 12]. Based on dietary guidance, the RFS included 13 vegetables, 6 fruits, 7 cereal products, 5 types of fish and seafood, 3 low-fat dairy products, nuts/almonds, and olive oil. When each recommended food item was consumed at least 1–3 times per month or more, 1 point was assigned. Participants could receive a maximum of 36 points, with a higher score representing better diet quality. The modified Non-RFS included 9 food items (we omitted 3 unprocessed and 4 processed red meat food items that were included in the original score), which are not recommended from a health point of view, i.e., 3 high-fat dairy products, white bread, sweets, potato chips/popcorn and fried potatoes/French fries, unprocessed offal, mayonnaise, and ice cream. Consumption of any of these non-recommended food items 3 times per week or more often was assigned 1 point, and if less often 0 points. Subjects could receive a maximum of 9 points, with the maximum points corresponding to the lowest diet quality [11].

Data on education, body weight, height, and alcohol consumption was collected in 1987–1990 and 1997, while smoking status and physical activity level was collected in 1997. For all covariates the data collected in 1997 was used in the analyses. Body mass index (BMI) was calculated by dividing weight (kg) by the square of height (m). Women were asked about the average number of cigarettes smoked per day at different ages (15–20, 21–30, 31–40, 41–50 and 51–60 years of age, and in the present year), the age when they started smoking, and how many years since they stopped smoking. Pack-years were estimated by multiplying the number of years smoked by the reported number of cigarettes smoked per day within the respective age groups. Total physical activity score, measured as metabolic equivalents (MET h/day), was calculated by multiplying time per day spent on five predefined activities (occupation, housework, walking or bicycling, and exercise as well as sedentary time) and the typical energy expenditure of these activities. All individual activities were summed to calculate the metabolic equivalent-hour per day (24-h) score [13, 14].

### Case ascertainment

With the use of the unique personal identification number assigned to each Swedish resident, we identified incident COPD cases through linkage with the Swedish Patient Register (in-patient and out-patient registers) and the Swedish Cause of Death Register. Events of COPD were defined according to the International Classification of Diseases and Related Health Problems, 10th Revision (ICD code J44). In the present study, a COPD case was defined as the first diagnosis of COPD in the Swedish Patient Register (listed either as the primary diagnosis or at any diagnosis position) or in the Cause of Death Register (only the primary position of COPD). It has been previously reported that from 1999 to 2009 (a time period overlapping with our study) an increasingly higher proportion of patients with COPD were detected and diagnosed each year (59% in 1999 vs. 81% in 2009) [15]. Patients that received a COPD diagnosis in 2009 were, on average, 7 years younger than those who were diagnosed in 1999 (66 vs. 73 years old) [15]. To examine an influence of a potential under-ascertainment during the early years of follow-up, we have analyzed the distribution of new annual COPD cases from 1998 to 2014 (Supplementary Fig. 1). During the first 4 years of follow-up (1998–2001), the number of diagnosis of COPD was significantly lower (38 cases of COPD per year) than after 2001 (114 cases of COPD per year); however, we did not observe that risk estimates for the first years of follow-up were attenuated in comparison to the later period (Supplementary Table 1). Despite this, we introduced a 4-year lag period from 1998, such that the study period for our main analysis started in 2002.

### Statistical analysis

Cox proportional hazards regression models were used to estimate hazard ratios (HRs) with 95% confidence intervals (CIs) of COPD risk by three arbitrary categories of unprocessed and processed red meat consumption (< 25, 25–49.9 and ≥ 50 g/day) and by 50 g/day-increment of consumption of processed red meat. Usage categorization simplifies the interpretation in relation to portion sizes (1 serving of processed meat = 50 g, and is the equivalent of one sausage or about two slices of cold cuts) and facilitates communication of results. We examined baseline (1997) as well as long-term (1987 and 1997) diet with the lowest category of red meat consumption as the reference group.

Multivariable models were adjusted for age (continuous, years), education (less than high school, high school, or university), smoking status and pack-years of smoking (never; past < 20, 20–39, ≥ 40 pack-years; current < 20, 20–39, ≥ 40 pack-years), BMI (< 18.5, 18.5–24.9, 25–29.9, ≥ 30 kg/m^2^), total physical activity (MET h/day, quintiles), alcohol consumption (g/day, quintiles), energy intake (kcal/day, quintiles), RFS (scores, continuous), and modified Non-RFS (scores, continuous). The purpose of including the two food scores in the multivariable models was to assess the associations between unprocessed and processed red meat consumption and risk of COPD independent of other dietary factors. Missing data on education (0.3%), smoking status (3.6%), BMI (1.6%), and total physical activity (21.1%) were modeled as separate categories. Results for unprocessed and processed red meat consumption were mutually adjusted through inclusion in the same multivariable model.

The proportional hazards assumption was met for all variables by regressing scaled Schoenfeld residuals against survival time. Tests for linear trend were performed using the median value of each exposure category of unprocessed and processed red meat consumption and modeling theses values as continuous variables. Using a likelihood ratio test, we tested statistical interactions on the multiplicative scale between unprocessed and processed red meat consumption in predicting incidence of COPD according to smoking status.

Statistical analyses were implement using SAS (version 9.4), and all two-sided *P* values ≤ 0.05 were considered statistically significant.

## Results

### Characteristics of the cohort

Baseline (1997) characteristics of the study population by categories of unprocessed and processed red meat consumption are presented in Table 1. Median consumption of unprocessed and processed red meat differed 4.2-fold and 3.7-fold, respectively, between the lowest and the highest categories. The Spearman’s correlation coefficient between unprocessed and processed red meat consumption was 0.32. In contrast to ex-smokers, women who were current smokers were more likely to be in the highest category of processed red meat consumption, and women with higher processed red meat consumption were less likely to have a university education than those with lower consumption. Moreover, women with higher consumption of unprocessed red meat were, on average, 6.6 years younger than those with lower consumption, and women with high consumption of both unprocessed and processed red meat had, on average, higher intake of energy and products from the non-recommended food list.

**Table 1:** Age-standardized characteristics of the Swedish mammography cohort by categories of unprocessed and processed red meat consumption in baseline, in the year 1997 (*n* = 34,053)

Characteristic	Unprocessed red meat, g/day (median)				Processed red meat, g/day (median)			
	< 25 (14.0)	25–49.9	≥ 50 (58.2)	*P* for trend^a^	< 25 (16.5)	25–49.9	≥ 50 (60.5)	*P* for trend^a^
No. of women	11,518	18,036	4499		16,500	13,403	4150	
Age, year	63.7 ± 9.0^b^	60.8 ± 8.6	57.1 ± 9.0	< 0.001	62.0 ± 9.1	60.3 ± 8.9	61.2 ± 8.9	< 0.001
Education, university, %	19.3	18.4	19.9	< 0.001	22.7	16.2	13.5	< 0.001
BMI, kg/m^2^	24.9 ± 3.9	25.0 ± 3.9	25.3 ± 4.1	0.001	24.8 ± 3.8	25.1 ± 3.9	25.5 ± 4.2	< 0.001
Total physical activity, MET × h/day	42.5 ± 4.9	42.4 ± 4.7	42.5 ± 4.9	0.005	42.2 ± 4.8	42.6 ± 4.7	42.9 ± 5.0	< 0.001
Smoking status, %				< 0.001				< 0.001
Current smokers	23.8	22.0	23.1		22.6	22.6	23.9	
Ex-smokers	23.0	22.7	21.8		23.6	22.6	19.8	
Never smokers	51.4	53.7	53.6		52.1	53.1	54.8	
Alcohol, g/day	3.8 ± 4.8	4.3 ± 5.2	4.5 ± 6.4	< 0.001	4.3 ± 5.4	4.2 ± 5.0	4.1 ± 5.3	0.02
Energy, kcal/day	1586 ± 489	1780 ± 474	2068 ± 627	< 0.001	1616 ± 488	1793 ± 468	2106 ± 615	< 0.001
RFS, score	24.5 ± 6.0	26.1 ± 4.9	25.0 ± 5.4	< 0.001	24.8 ± 5.7	26.1 ± 5.0	25.6 ± 5.4	< 0.001
Modified Non-RFS, score	2.9 ± 1.1	3.1 ± 1.1	3.3 ± 1.2	< 0.001	2.9 ± 1.1	3.2 ± 1.1	3.3 ± 1.1	< 0.001

*BMI* body mass index, *MET* metabolic equivalent of energy expenditure (kcal/kg × h), *RFS* Recommended Food Score, *Non-RFS* Non-Recommended Food Score

^a^*P* values were calculated across categories of red meat consumption by using age-adjusted linear models for continuous variable and Pearson’s Chi-square for categorized variables

^b^Mean ± SD (all such values)

### Long-term red meat consumption and COPD

During a mean of 11.6 years of follow-up (393,831 person-years, 2002–2014), we ascertained 1488 incident cases of COPD diagnosis. Unprocessed and processed red meat consumption assessed at baseline (1997) was not associated with COPD incidence (Table 2). The HRs (95% CI) for women in the highest category (≥ 50 g/day) compared to those in the lowest category (< 25 g/day) were 0.89 (95% CI 0.74–1.07) for unprocessed and 1.16 (95% CI 0.98–1.36) for processed red meat consumption. Taking into consideration the long-term consumption of unprocessed and processed red meat, reflecting the average consumption at baseline and 10 years prior to baseline (i.e., 1987 and 1997 years), women in the highest category of processed red meat consumption compared with the lowest category had a 36% (95% CI 3–79%) higher risk of COPD, but there was no significant linear trend (*P* for trend = 0.32). We observed an increasing risk of COPD only among women who consumed 50 g or more per day of processed meat. No statistically significant association was observed between long-term unprocessed red meat consumption and COPD incidence (HR = 0.87; 95% CI 0.74–1.02).

**Table 2:** Hazard ratios (95% CIs) of chronic obstructive pulmonary disease during follow-up (2002–2014) of 34 053 Swedish women by categories of unprocessed and processed red meat consumption

	Categories			*P* for trend
Unprocessed red meat, g/day (median)	< 25 (14.0)	25–49.9	≥ 50 (58.2)	
No. of cases/person-years	579/128 918	739/211 535	170/53 378	
Age-adjusted SIR	448	377	415	
Age- and smoking adjusted HR (1997)	1.00	0.87 (0.78–0.97)	0.91 (0.76–1.08)	0.06
Multivariable HR^a,b^				
Baseline diet (1997)	1.00	0.93 (0.82–1.04)	0.89 (0.74–1.07)	0.15
Long-term diet (1987 and 1997)	1.00	0.90 (0.79–1.03)	0.87 (0.74–1.02)	0.11
Processed red meat, g/day (median)	< 25 (16.5)	25.0–49.9	≥ 50 (60.5)	
No. of cases/person-years	741 / 188 890	537 / 157 552	210 / 47 389	
Age-adjusted SIR	413	383	477	
Age- and smoking adjusted HR (1997)	1.00	0.92 (0.82–1.02)	1.18 (1.01–1.37)	0.19
Multivariable HR^a,b^				
Baseline diet (1997)	1.00	0.96 (0.85–1.08)	1.16 (0.98–1.36)	0.19
Long-term diet (1987 and 1997)	1.00	0.95 (0.84–1.07)	1.36 (1.03–1.79)	0.32

*CI* confidence interval, *HR* hazard ratio, *SIR* standardized incidence rate per 100,000

^a^Adjusted for age (years, continuous), education (less than high school, high school, or university), BMI (< 18.5, 18.5–24.9, 25–29.9, or ≥ 30 kg/m^2^), total physical activity (MET × h/day, quintiles), smoking status and pack-years of smoking (never; past < 20, 20–39, or ≥ 40 pack-years; or current < 20, 20–39, or ≥ 40 pack-years), alcohol consumption (g/day, quintiles), intake of energy (kcal/day, quintiles), and Recommended Food Score (scores, continuous) and modified Non-Recommended Food Score (scores, continuous)

^b^Unprocessed and processed red meat were included in the same multivariable model

Although, we did not observe a statistically significant interaction between long-term processed red meat consumption and smoking status in relation to risk of COPD (*P* value for interaction = 0.30), we stratified results by smoking status due to that it is a main risk factor of COPD development (Table 3). There was no significant association between COPD and baseline meat consumption in ever, current, or never smokers, but there was in ex-smokers. Long-term processed red meat consumption was associated with both ever and ex-smokers, but not current or never smokers. Ex-smokers women with long-term processed red meat consumption ≥ 50 g/day compared to those with consumption < 25 g/day had a 2.3-fold (95% CI 1.24–4.12, *P* trend = 0.016) higher risk of COPD, while in current smokers the HR was 1.26 (95% CI 0.90–1.76). Among ex-smokers, each 50 g/day processed red meat consumption was associated with an additional 46% (95% CI 6–99%) higher COPD risk. A statistically significant association was not observed between unprocessed red meat consumption and smoking status in relation to COPD risk (*P* value for interaction = 0.12); the results after stratification by smoking status are presented in Supplementary Table 2.

**Table 3:** Hazard ratios (95% CIs) of chronic obstructive pulmonary disease during follow-up (2002–2014) of Swedish women by categories of processed red meat consumption and smoking status

	Categories of processed red meat consumption, g/day (median)			*P* for trend
	< 25 (16.5)	25–49.9	≥ 50 (60.5)	
Ever smokers				
No. of cases/person-years	642/85,260	460/73,016	190/20,790	
Age-adjusted SIR	923	847	1202	
Baseline diet (1997)^a,b^	1.00	0.92 (0.82–1.05)	1.17 (0.98–1.39)	0.22
Long-term diet HR (1987 and 1997)^a,b^	1.00	0.92 (0.81–1.05)	1.41 (1.05–1.89)	0.42
Current smokers				
No. of cases/person-years	489/40,067	348/35,259	138/11,029	
Age-adjusted SIR	1483	1289	1606	
Baseline diet (1997)^a,b^	1.00	0.88 (0.76–1.02)	1.01 (0.82–1.23)	0.70
Long-term diet HR (1987 and 1997)^a,b^	1.00	0.85 (0.73–0.98)	1.26 (0.90–1.76)	0.72
Ex-smokers				
No. of cases/person-years	153/45,193	112/37,757	52/9761	
Age-adjusted SIR	432	448	755	
Baseline diet (1997)^a,b^	1.00	1.02 (0.79–1.32)	1.78 (1.26–2.51)	0.004
Long-term diet HR (1987 and 1997)^a,b^	1.00	1.15 (0.89–1.49)	2.26 (1.24–4.12)	0.016
Never smokers				
No. of cases/person-years	89/100,407	72/81,996	20/25,869	
Age-adjusted SIR	81	93	82	
Baseline diet (1997)^a^	1.00	1.27 (0.92–1.77)	1.08 (0.65–1.81)	0.48
Long-term diet HR (1987 and 1997)^a^	1.00	1.21 (0.86–1.70)	1.13 (0.48–2.65)	0.39

*CI* confidence interval, *HR* hazard ratio, *SIR* standardized incidence rate per 100,000

^a^Adjusted for age (years, continuous), education (less than high school, high school, or university), BMI (< 18.5, 18.5–24.9, 25–29.9, ≥ 30 kg/m^2^), total physical activity (MET × h/day, quintiles), alcohol consumption (g/day, quintiles), intake of energy (kcal/day, quintiles), Recommended Food Score (scores, continuous), modified Non-Recommended Food Score (scores, continuous), and consumption of unprocessed red meat (categories: < 25, 25–44.9, ≥ 50 g/day)

^b^Additionally adjusted for pack-years of smoking (< 20, 20–39, or ≥ 40 pack-years)

## Discussion

In this large population-based prospective cohort of women, baseline and especially long-term consumption of processed red meat were associated with higher risk of COPD. Women with long-term processed meat consumption ≥ 50 g/day compared to those who consumed < 25 g/day of these products had a 36% increased risk of COPD; this association was confined to ex-smokers who had a 2.3-fold increased risk. We did not find similar associations for long-term consumption of unprocessed red meat.

Results of this study are consistent with our findings in the Cohort of Swedish Men [11], with two earlier published prospective studies conducted among men [10] and women [9] in the United States, as well as with two cross-sectional studies [16, 17]. In each of these studies positive associations between processed red meat consumption and increased risk of COPD was observed [9–11, 16, 17]. Specifically, in the cross-sectional studies inverse associations were observed between processed meat consumption and lung function including forced expiratory volume in 1 s (FEV_1_) [16, 17], forced vital capacity (FVC) [16] and FEV1/FVC ratio [16, 17]. Moreover, in our previous study among men [11], we observed a statistically significant interaction between smoking status and processed red meat consumption in relation to risk of COPD. In the current study, the increased risk of COPD was confined to ex-smokers, while among the Cohort of Swedish Men this increased risk was limited to the current smokers [11]. We can speculate that this difference may be a result of sex-specific pathogenic mechanisms, sex hormones, or different baseline health status between men and women. In our study, we did not find the statistically significant interaction between smoking status and consumption of processed red meat in relation to risk of COPD (*P* value for interaction = 0.30). Similarly in the Health Professionals Follow-up Study [10] and the Nurses’ Health Study [9], there was no statistically significant interaction between smoking status and consumption of processed red meat in relation to risk of COPD; however, the stratification analysis showed associations similar to those found among our cohorts of Swedish men and women. Among US women, the HR of COPD in ex-smokers compared to current smokers for the highest vs. the lowest processed red meat consumption was also higher; i.e., HR was 2.34 (95% CI 0.98–5.62) for ex-smokers and 1.30 (95% CI 0.77–2.20) for current smokers [9]. It should be noted that women in the cohort had rather low smoking intensity, only 275 women ever smoked more than 20 cigarettes per day during one’s smoking lifetime. Moreover, percentage of women smoking 20 or more cigarettes per day was higher among ex-smokers (6.1%) than among current smokers (4.6%). It may at least partly explain that the association between processed red meat consumption and COPD incidence was observed mainly in ex-smokers.

We did not observe an association between processed red meat consumption and risk of COPD in never smokers; however, the number of COPD cases was small and statistical power of the analysis was limited.

It is known that the pathogenesis of COPD is connected to oxidative stress and inflammatory processes in lung cells, which are particularly intense among smokers [18]. Cigarette smoke contains many toxic chemical compounds and is a major source of nitrites generating the formation of reactive nitrogen species that are likely to damage lung and vascular cells in such a way that it contributes to, or perpetuates, the development of COPD. Specifically, a high production of reactive nitrogen species, such as peroxynitrite, can promote inflammatory processes causing DNA damage, protein modification, and cell dysfunction by inhibiting mitochondrial respiration [18]. We hypothesize that the harmful effect of consumption of processed red meat is caused by additional amounts of nitrites contained in these products. Nitrites are added to processed meat during the manufacturing process as a preservative, antimicrobial agent, and color fixative [19]. Thus, smokers with high consumption of processed meat are more likely to be exposed to the pro-inflammatory and pro-oxidant effects of nitrites than those with low processed meat consumption. Some studies suggest that systemic inflammatory response to smoking in COPD may be different in men and women [20]. In a cross-sectional study, current smokers who were women compared to men had a higher extent of oxidative DNA and lipid damage despite higher serum levels of fat-soluble antioxidants [21]; however, it remains unknown to what extent there are sex differences in terms of the capacity to handle oxidative stress.

Among the strengths of the current study, we would like to emphasize the population-based prospective design, large number of total incident COPD cases (although only 181 among never smokers), and detailed information on long-term diet. In addition, the available data on potential risk factors for COPD allowed us to extensively adjust for confounders. Adjusted models for RFS and modified Non-RFS make results more likely independent of dietary factors other than red meat consumption. However, unmeasured or residual confounding cannot be disregarded; especially that smoking status may influence reporting [22]. Although the FFQ used in this study had a relatively high validity for the consumption of individual red meat and processed meat food items/categories, some misclassification of unprocessed and processed red meat consumption is probable. Because of the prospective design, any misclassification of processed and unprocessed red meat consumption would be non-differential and would most likely have attenuated rather than exaggerated the true associations. Finally, although we used a lag analysis to delay the start of follow-up by approximately 4 years, we cannot rule out some under-ascertainment of COPD. However, we observed that risk estimates for the first years of follow-up were not attenuated in comparison to the later period. Moreover, we cannot rule out that some patients classified as having COPD had obtained the diagnosis without the correct spirometry assessment, even though this investigation is formally required for the correct diagnosis of COPD. Because of this, we believe that future studies in this particular research area should include a spirometry assessment verifying true COPD as this would further strengthen the validity of the data. Presumably, such new prospective studies will also benefit from the fact that a higher proportion of patents with COPD are now being identified, compared to what the case was at the turn of the century [15].

Our findings are consistent with previously published results of prospective studies indicating that processed red meat consumption should be limited not only for CVD and cancer prevention, but also because of its association with the development of COPD among smokers. Although our results indicate that diet may influence COPD risk, the most important public health message is still smoking cessation.

## Electronic supplementary material

Below is the link to the electronic supplementary material.

Supplementary material 1 (DOCX 39 KB)

## References

[1] World Health Organization. Cause-specific mortality. Estimates for 2000–2012. Health statistics and information systems. http://www.who.int/healthinfo/global_burden_disease/estimates/en/index1.html. Accessed 05 Dec 2016

[2] Wolk A (2017). Potential health hazards of eating red meat. J Intern Med.

[3] Larsson SC, Orsini N (2014). Red meat and processed meat consumption and all-cause mortality: a meta-analysis. Am J Epidemiol.

[4] Pan A, Sun Q, Bernstein AM, Schulze MB, Manson JE, Stampfer MJ (2012). Red meat consumption and mortality: results from 2 prospective cohort studies. Arch Intern Med.

[5] Kaluza J, Wolk A, Larsson SC (2012). Red meat consumption and risk of stroke: a meta-analysis of prospective studies. Stroke.

[6] Larsson SC, Wolk A (2012). Red and processed meat consumption and risk of pancreatic cancer: meta-analysis of prospective studies. Br J Cancer.

[7] Chan DSM, Lau R, Aune D, Vieira R, Greenwood DC, Kampman E (2011). Red and processed meat and colorectal cancer incidence: meta-analysis of prospective studies. PLoS One.

[8] Feskens EJM, Sluik D, van Woudenbergh GJ (2013). Meat consumption, diabetes, and its complications. Curr Diabetes Rep.

[9] Jiang R, Camargo CA, Varraso R, Paik DC, Willett WC, Barr RG (2008). Consumption of cured meats and prospective risk of chronic obstructive pulmonary disease in women. Am J Clin Nutr.

[10] Varraso R, Jiang R, Barr RG, Willett WC, Camargo CA (2007). Prospective study of cured meats consumption and risk of chronic obstructive pulmonary disease in men. Am J Epidemiol.

[11] Kaluza J, Larsson SC, Linden A, Wolk A (2016). Consumption of unprocessed and processed red meat and the risk of chronic obstructive pulmonary disease: a prospective cohort study of men. Am J Epidemiol.

[12] Kaluza J, Håkansson N, Brzozowska A, Wolk A (2009). Diet quality and mortality: a population-based prospective study of men. Eur J Clin Nutr.

[13] Norman A, Bellocco R, Bergström A, Wolk A (2001). Validity and reproducibility of self-reported total physical activity—differences by relative weight. Int J Obes.

[14] Orsini N, Bellocco R, Bottai M, Hagströmer M, Sjöström M, Pagano M (2008). Validity of self-reported total physical activity questionnaire among older women. Eur J Epidemiol.

[15] Ställberg B, Janson C, Johansson G, Larsson K, Stratelis G, Telg G (2013). Management, morbidity and mortality of COPD during an 11-year period: an observational retrospective epidemiological register study in Sweden (PATHOS). Prim Care Respir J.

[16] Okubo H, Shaheen SO, Ntani G, Jameson KA, Syddall HE, Aihie Sayer A (2014). Processed meat consumption and lung function: modification by antioxidants and smoking. Eur Respir J.

[17] Jiang R, Paik DC, Hankinson JL, Barr RG (2007). Cured meat consumption, lung function, and chronic obstructive pulmonary disease among united states adults. Am J Respir Crit Care Med.

[18] Ricciardolo FLM, Di Stefano A, Sabatini F, Folkerts G (2006). Reactive nitrogen species in the respiratory tract. Eur J Pharmacol.

[19] Walters C (1991). Nitrate and nitrite in food. Nitrates and nitrites in food and water.

[20] Faner R, Gonzalez N, Cruz T, Kalko SG, Agustí A (2014). Systemic inflammatory response to smoking in chronic obstructive pulmonary disease: evidence of a gender effect. PLoS One.

[21] Hakim IA, Harris R, Garland L, Cordova CA, Mikhael DM, Sherry Chow H-H (2012). Gender difference in systemic oxidative stress and antioxidant capacity in current and former heavy smokers. Cancer Epidemiol Biomarkers Prev.

[22] Lopes TS, Luiz RR, Hoffman DJ, Ferriolli E, Pfrimer K, Moura AS (2016). Misreport of energy intake assessed with food records and 24-h recalls compared with total energy expenditure estimated with DLW. Eur J Clin Nutr.

