# Supplementary material for: Long-term unprocessed and processed red meat consumption and risk of chronic obstructive pulmonary disease: a prospective cohort study of women
Source: Eur J Nutr. 2018 Mar 12;58(2):665–72. doi: 10.1007/s00394-018-1658-5 (PMC6437121; doi:10.1007/s00394-018-1658-5)
Supplement: Supplementary file 1 — Supplementary material 1 (DOCX 39 KB) [file 394_2018_1658_MOESM1_ESM.docx]

**Supplementary Material**

Long-term unprocessed and processed red meat consumption and risk of chronic obstructive pulmonary disease: A prospective cohort study of women

European Journal of Nutrition

Joanna Kaluza^*^, Holly Harris, Anders Linden, Alicja Wolk

^*^e-mail: joanna_kaluza@sggw.pl

**Start of follow-up**

The follow-up period for this study began on January 1, 2002 and ended December 31, 2014. Based on the distribution of new annual COPD cases from 1998 to 2014 (Supplementary Fig. 1), we delayed the start of follow-up approximately 4 years from study baseline (1997) due to under-diagnosis of COPD cases in the first 4 years. During the first four years of follow-up (1998-2001), the number of COPD cases diagnosed was significantly lower (mean: 38 cases of COPD per year) than after 2001 (mean: 114 cases of COPD per year).

To examine an impact of a potential under-diagnosis during the early years of follow-up we calculated HRs stratified by time of diagnosis – Supplementary Table 1. Although, we observed under-diagnosis in 1998-2001, the HR after excluding the first four years of follow-up, was similar to those obtained for entire follow-up period (1998-2014).

(year)

(n)

**Supplementary Fig. 1.** Frequency of new diagnosis of COPD cases identified through the Swedish Patient Register between 1998 and 2014.

**Supplementary Table 1.** Hazard Ratios (95% CIs) of Chronic Obstructive Pulmonary Disease by Categories of Long-term Processed Red Meat Consumption Stratified by Time of Diagnosis in 34 053 Swedish Women, 1998 to 2014

| Time of COPD diagnose, years | Long-term processed red meat consumption, g/day (median) | | | | *P* for trend |
| --- | --- | --- | --- | --- | --- |
|  | < 25 (16.5) | | 25 – 49.9 | ≥ 50 (60.5) |  |
| ***Follow-up 1998–2014 (17years)*** | | |  |  |  |
| Multivariable HR^a^ | | 1.00 | 0.95 (0.85-1.07) | 1.36 (1.05-1.76) | 0.28 |
| ***Follow-up 1998–2001(4 years)*** | |  |  |  |  |
| Multivariable HR^a^ | | 1.00 | 0.99 (0.67-1.45) | 1.44 (0.66-3.15) | 0.52 |
| ***Follow-up 2002–2014 (13 years)*** | | |  |  |  |
| Multivariable HR^a^ | | 1.00 | 0.95 (0.84-1.07) | 1.36 (1.03-1.79) | 0.32 |

Abbreviations: CI, confidence interval; HR, hazard ratio.

^a^Adjusted for age (years, continuous), education (less than high school, high school, or university), BMI (<18.5, 18.5-24.9, 25-29.9, or ≥30 kg/m2), total physical activity (MET×h/day, quintiles), smoking status and pack-years of smoking (never; past <20, 20-39, or ≥40 pack-years; or current <20, 20-39, or ≥40 pack-years), alcohol consumption (g/day, quintiles), intake of energy (kcal/day, quintiles), Recommended Food Score (scores, continuous), modified Non-Recommended Food Score (scores, continuous), and long-term unprocessed red meat consumption (<25, 25-49.9, or ≥50 g/day).

**Supplementary Table 2.** Hazard Ratios (95% CIs) of Chronic Obstructive Pulmonary Disease during Follow-up (2002-2014) of Swedish Women by Categories of Unprocessed Red Meat Consumption and Smoking Status

|  | Categories of unprocessed red meat consumption, g/d (median) | | | *P* for trend | | |
| --- | --- | --- | --- | --- | --- | --- |
|  | < 25 (14.0) | 25 – 49.9 | ≥ 50 (58.2) |  |  |  |
| *Ever smokers* |  |  |  |  |  | |
| No. of cases / Person-years | 490 / 55 905 | 648 / 95 662 | 154 / 27 499 |  | | |
| Age-adjusted SIR | 940 | 796 | 969 |  | | |
| Baseline diet (1997)^a,b^ | 1.00 | 0.94 (0.83-1.07) | 0.91 (0.75-1.11) | 0.28 | | |
| Long-term diet HR (1987 and 1997)^a,b^ | 1.00 | 0.93 (0.81-1.08) | 0.88 (0.74-1.05) | 0.17 | | |
| *Current smokers* | | | | | | |
| No. of cases / Person-years | 364 / 27 182 | 489 / 45 463 | 122 / 13 710 |  | | |
| Age-adjusted SIR | 1 428 | 1 250 | 1 483 |  | | |
| Baseline diet (1997)^a,b^ | 1.00 | 0.97 (0.84-1.12) | 0.98 (0.78-1.22) | 0.76 | | |
| Long-term diet HR (1987 and 1997)^a,b^ | 1.00 | 1.00 (0.85-1.18) | 0.91 (0.74-1.12) | 0.31 | | |
| *Ex-smokers* |  |  |  |  |  |  |
| No. of cases / Person-years | 126 / 28 724 | 159 / 50 199 | 32 / 13 789 |  | | |
| Age-adjusted SIR | 481 | 399 | 476 |  | | |
| Baseline diet (1997)^a,b^ | 1.00 | 0.88 (0.69-1.13) | 0.75 (0.50-1.14) | 0.15 | | |
| Long-term diet HR (1987 and 1997)^a,b^ | 1.00 | 0.77 (0.59-1.02) | 0.81 (0.57-1.15) | 0.30 | | |
| *Never smokers* |  |  |  |  |  |  |
| No. of cases / Person-years | 81 / 70 586 | 85 / 112 512 | 15 / 25 174 |  | | |
| Age-adjusted SIR | 92 | 74 | 66 |  | | |
| Baseline diet (1997)^a^ | 1.00 | 0.85 (0.61-1.17) | 0.76 (0.43-1.36) | 0.25 | | |
| Long-term diet HR (1987 and 1997)^a^ | 1.00 | 0.80 (0.57-1.13) | 0.84 (0.52-1.34) | 0.43 | | |

CI: confidence interval, HR: hazard ratio, SIR: standardized incidence rate per 100,000.

^a^Adjusted for age (years, continuous), education (less than high school, high school, or university), BMI (<18.5, 18.5-24.9, 25-29.9, ≥30 kg/m2), total physical activity (MET×h/day, quintiles), alcohol consumption (g/day, quintiles), intake of energy (kcal/day, quintiles), Recommended Food Score (scores, continuous), modified Non-Recommended Food Score (scores, continuous), and consumption of unprocessed red meat (categories: <25, 25-44.9, ≥ 50 g/day).

^b^Additionally adjusted for pack-years of smoking (<20, 20-39, or ≥40 pack-years).
